# Genome Sequence, Comparative Genome Analysis, and Expression Profiling of the Chitinase GH18 Gene Family in *Cordyceps javanica* Bd01

**DOI:** 10.3390/ijms26052031

**Published:** 2025-02-26

**Authors:** Tao Zhu, Mehboob Hussain, Jingyi Ning, Xiao Chen, Chunlan Shi, Dewei Yang, Xi Gao, Guoxing Wu

**Affiliations:** State Key Laboratory for Conservation and Utilization of Bio-Resources in Yunnan, Yunnan Agricultural University, Kunming 650201, China; xiansenrobin@gmail.com (T.Z.); mehboobhussain413@gmail.com (M.H.); ynnjingyi@163.com (J.N.); 15108646682@163.com (X.C.); shiclan22@163.com (C.S.); yingydw@163.com (D.Y.); chonchon@163.com (X.G.)

**Keywords:** *Cordyceps javanica*, GH18, phylogenetic, conservatism

## Abstract

The fungus *Cordyceps javanica* is known for entomopathogenicity and effective in the control of various arthropods. Here, we aimed to reveal the chitinase GH18 gene family expansion through the high throughput sequencing of the genome of *C. javanica* strain Bd01 isolated from *Xylotrechus quadripes* larvae. The genome was 34 Mb in size with 9590 protein-coding genes. By comparative genome analysis, it was found that the family GH18 of chitinase genes was expanded in *C. javanica* Bd01. The phylogenetic analysis of 27 GH18 genes, compared with those from four other species, revealed that the genes could be categorized into three distinct groups based on their conserved domains. Genes within the same cluster exhibited shared protein motifs and orthologous relationships. The molecular mass of these GH18 genes ranged from 14.03 kDa to 81.41 kDa, while their theoretical isoelectric point (pI) values spanned from 4.40 to 7.92. Most chitinases were characterized as extracellular, hydrophilic, and thermostable proteins with a negative charge. Additionally, they demonstrated favorable in vivo half-life stability. A three-dimensional structural model of the GH18 protein was further generated using the SWISS-MODEL server. These findings establish a robust genomic framework elucidating the functional diversity, evolutionary conservation patterns, and mechanistic contributions of virulence-associated genetic determinants.

## 1. Introduction

Entomopathogenic fungi (EPF), constituting the most diverse group of arthropod-pathogenic microorganisms, exhibit expansive host tropism and represent the oldest class of biocontrol agents employed in integrated pest management (IPM). *Cordyceps javanica*, a hypocrealean ascomycete within the family *Cordycipitaceae* (*Ascomycota: Sordariomycetes*) [1], has demonstrated broad-spectrum insecticidal efficacy across 11 arthropod species spanning 10 genera. Bioassays confirm its virulence against the following organisms: (1) *Hemiptera*: 100% mortality in Asian citrus psyllid (*Diaphorina citri*) [2], high efficacy against whiteflies (*Bemisia tabaci* [3], *B. argentifolii* [4]), aphid control (*Hyalopterus pruni*, *Aphis pomi*) [5], and tea green leafhopper (*Empoasca vitis*) [6]; (2) *Thysanoptera*: melon thrips (*Thrips palmi*) [7]; (3) *Hymenoptera*: red imported fire ant (*Solenopsis invicta*) [8]; (4) *Isoptera*: subterranean termite (*Coptotermes gestroi*) [9]; and (5) *Lepidoptera*: cabbage moth (*Mamestra brassicae*) [10] and Gypsy moth (*Lymantria dispar*) [11]. These findings position *C. javanica* as a phylogenetically versatile entomopathogen with a cross-order pathogenic capacity, underscoring its potential for development as a multi-target biocontrol agent.

Entomopathogenic fungi establish pathogenesis through cuticular adhesion mediated by secreted hydrolytic enzymes, principally chitinases, proteases, and lipases, which serve as central virulence factors governing their pathogenic potential toward insect hosts [12,13,14,15,16]. Fungal chitinases exhibit dual functionality: they orchestrate critical developmental processes (sporulation, cytokinesis, and morphogenesis) and facilitate the host–pathogen interplay through cuticle degradation [17,18,19,20]. Phylogenetic classification based on conserved catalytic domains segregates chitinases into two structurally distinct families—glycoside hydrolase 18 (GH18) and GH19—as defined by the CAZy database [21]. The GH18 family demonstrates a phylum-transcendent conservation, ubiquitously distributed across bacterial, fungal, viral, metazoan, and embryophytic lineages [22,23,24,25]. Mechanistically, GH18 enzymes mediate the hydrolytic cleavage of β-1,4-glycosidic bonds in chitin polymers and participate in glycoprotein deglycosylation, underscoring their catalytic versatility [26,27]. To date, 75 chitinases from entomopathogenic fungi—including *Metarhizium anisopliae*, *Beauveria bassiana*, and *Isaria fumosorosea*—have been functionally characterized. Notably, the *chi2* and *chi3* genes isolated from *M. anisopliae* demonstrated critical roles in host infection processes during fungal pathogenesis [28,29]. Gene overexpression studies revealed that *Bbchit1* in *B. bassiana* significantly enhanced the species’ virulence against aphid hosts [30]. The functional analyses of *I. fumosorosea* chitinases further demonstrated that *Ifchit1* participated in both vegetative growth and virulence expression, while *Ifu-chit2* is hypothesized to mediate initial host–pathogen interactions during the early stages of infection [13,31,32]. The molecular machinery underlying chitinolytic activity and entomopathogenicity-associated genetic determinants in *C. javanica* bd01 remains poorly characterized. Notably, empirical data elucidating the tertiary conformations, catalytic mechanisms, and structure–function relationships of its chitinolytic apparatus remain conspicuously absent from the contemporary mycological literature.

This study elucidates the evolutionary trajectory of the GH18 chitinase gene family through the high-fidelity genome sequencing and hybrid assembly of *C. javanica* Bd01, an entomopathogen isolated from *Xylotrechus quadripes* larvae. Employing a comparative genomics framework, we systematically analyzed the Bd01 genome against phylogenetically diverse entomopathogenic fungi. An integrated phylogenomic–structural approach was implemented to resolve the following:

1. Evolutionary relationships: time-calibrated phylogeny of GH18 orthologs;
2. Domain architecture: conserved catalytic (TIM-barrel) and ancillary domains;
3. Signature motifs: MEME-identified chitin-binding and processivity motifs;
4. 3D structural dynamics: homology modeling of substrate-binding clefts.

These findings establish a genomic blueprint for fungal entomopathogenicity, revealing mechanistic insights into chitinolytic adaptation. The annotated GH18 repertoire serves as a functional scaffold for rational protein engineering and the targeted exploration of host–pathogen molecular interplay.

## 2. Results

### 2.1. Genome Sequencing, Assembly, and Annotation

**Figure 1:**
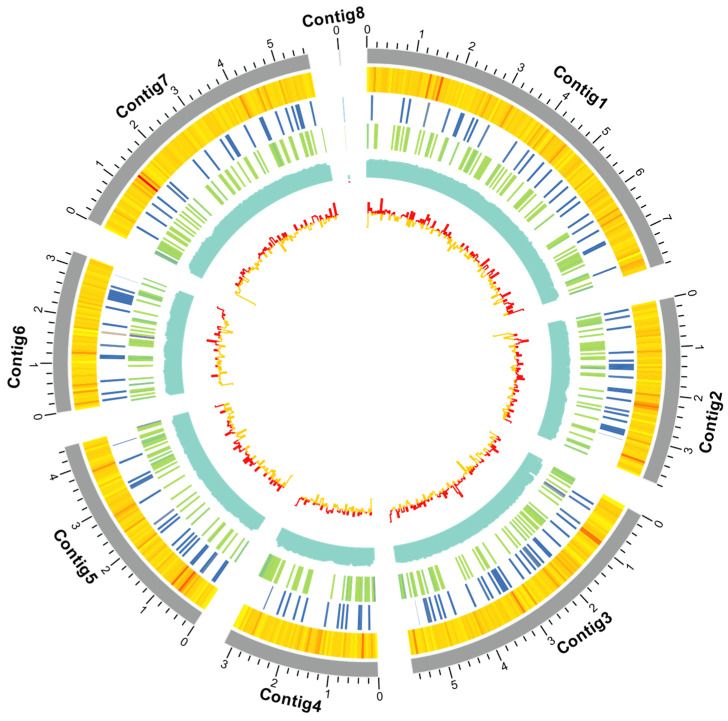
Circos map of the 8 contigs for *Cordyceps javanica* strain Bd01. Note: From inside to outside: gene density, non-coding RNA density, repetitive sequence coverage, GC percentage, and GC skew.

The hybrid genome assembly of *C*. *javanica* Bd01, generated via Illumina NovaSeq 6000 sequencing (2 × 150 bp), yielded 78.1 million high-quality reads (Q30 ≥ 92.4%), corresponding to ~229.6× coverage. The final assembly (NCBI BioProject accession: PRJNA1203233) comprises eight contigs (N50: 5.4 Mb) with 53.18% GC content, exhibiting 98.28% completeness as per BUSCO v5 analysis (ascomycota_odb10). The genome annotation identified 9590 protein-coding genes (gene density: 12.3 genes/Mb), 43 rRNA loci, and 131 tRNA genes, with repetitive elements constituting merely 1.04% (0.34 Mb) of the 33.8 Mb genome (Figure 1; Table 1).

**Figure 2:**
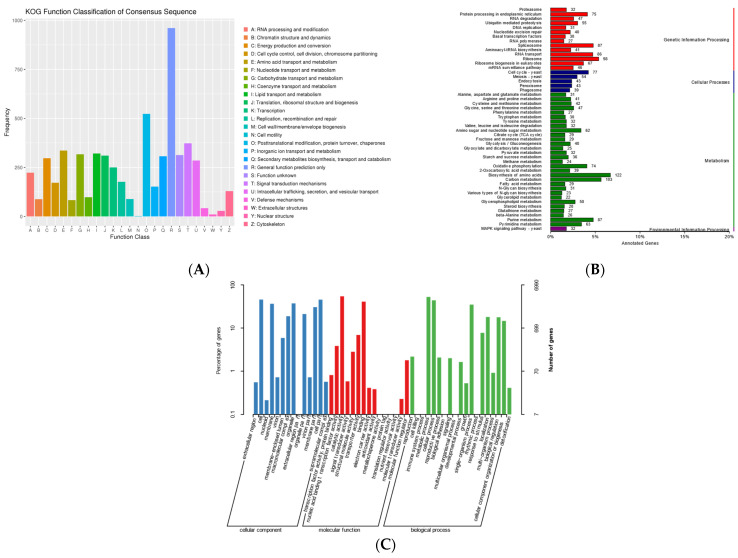
(**A**) KOG functional annotation classification of *Cordyceps javanica* Bd01; (**B**) classification statistics of KEGG annotations of *Cordyceps javanica* Bd01; (**C**) statistics of GO annotation classification of *Cordyceps javanica* Bd01. Note: The *x*-axis denotes the KOG functional classification categories, while the *y*-axis represents gene counts per category. Disproportionate gene distribution across functional groups reflects organism-specific metabolic prioritization and physiological adaptations, which are interpretable through comparative analysis of species-dependent enrichment patterns within each functional domain.

The functional annotation of 9590 proteins was performed using the InterProScan platform to identify the Pfam domains. The subsequent eukaryotic orthologous group (KOG) classification assigned 5236 proteins to distinct functional categories (Supplementary Table S1; Figure 2A), with metabolic processes representing the most abundant category at approximately 50% among the four major classifications.

The KEGG pathway mapping annotated 3111 genes (32.7%), distributed across four principal pathways: metabolism (41.2%), genetic information processing (24.5%), cellular processes (8.2%), and environmental information processing (1%) (Supplementary Table S2; Figure 2B). The Gene Ontology (GO) analysis categorized 6990 genes (73.7%) into three functional domains: molecular functions (51.1%), cellular components (30.6%), and biological processes (18.3%). Notably, 35 genes were associated with secondary metabolite biosynthetic processes (Supplementary Table S3; Figure 2C), while 1096 genes remained functionally uncharacterized across the KOG, GO, and KEGG databases.

### 2.2. Comparative Genomic Analysis

**Figure 3:**
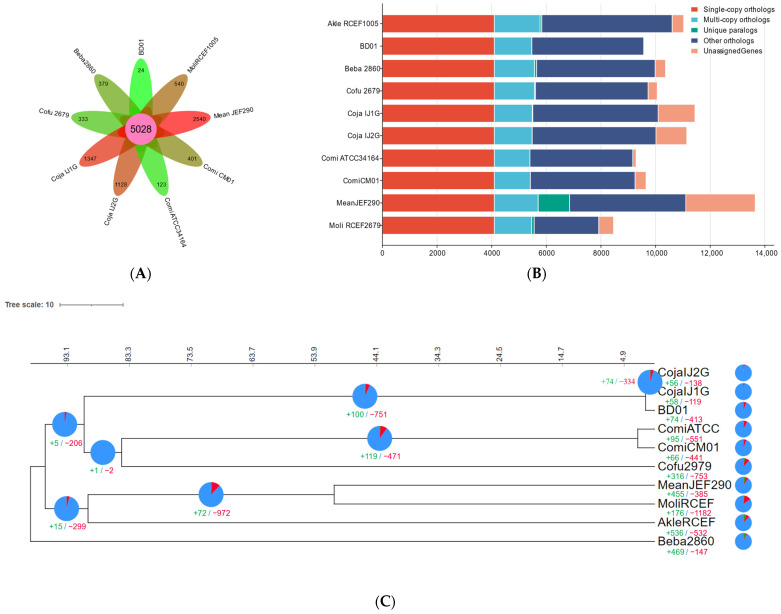

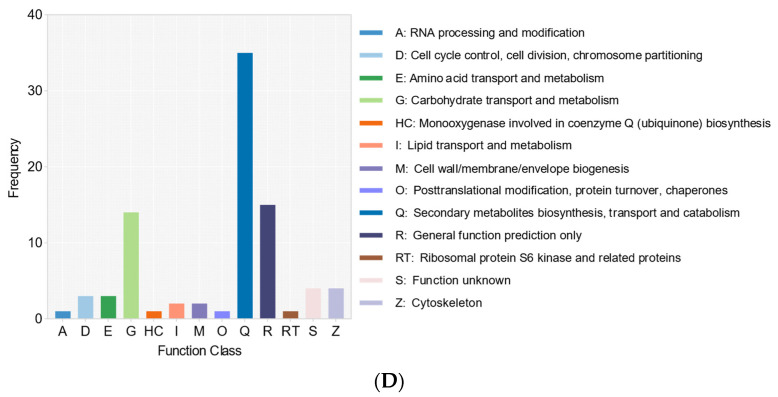
(**A**,**B**) Comparative analysis of homologous genomes; (**C**) phylogenetic tree of *Cordyceps javanica* Bd01 with different strains; (**D**) KOG functional annotation classification of expansion genes in *Cordyceps javanica* Bd01. Note: Divergence time calibration was implemented using two fossil constraints sourced from TimeTree: *Moelleriella libera* vs. *Metarhizium anisopliae* (37.3–118.4 million years (Ma), corresponding to the divergence of Hypocreales entomopathogens) and *Cordyceps militaris* vs. *Cordyceps fumosorosea* (96.4–325.5 Ma, reflecting the basal split within the *Cordycipitaceae* crown group). Calibration points were enforced as lognormal priors in MCMCTree (PAML v4.9), with soft bounds accommodating fossil age uncertainty. Scale bar indicates substitutions per site. The green and red numbers in the evolutionary tree represent the number of expansion genes and contraction genes at each node, respectively.

To investigate genome evolutionary dynamics in *C. javanica* Bd01 and its phylogenetically related entomopathogenic fungal strains, we analyzed lineage-specific genomic diversification mechanisms. This included profiling evolutionary changes through gene family expansion/contraction events and identifying lineage-restricted gene duplication/deletion patterns within homologous gene clusters. By OrthoFinder analysis, among the ten fungal genomes (*C. javanica* Bd01, *C. javanica* IJ1, *C. javanica* IJ2, *C. fumosorosea* ARSEF2679, *C. militaris* CM01, *C. militaris* ATCC34164, *Akanthomyces lecanii* RCEF1005, *B. bassiana* ARSEF2860, *M. libera* RCEF2490, and *M. anisopliae* JEF290), the OrthoFinder analysis identified 11,781 orthologous clusters across the ten fungal genomes, with 5028 (42.68%) conserved across all the strains (Figure 3A,B), demonstrating a significant phylogenetic congruence indicative of shared evolutionary ancestry.

Leveraging the amino acid sequences derived from 4096 single-copy orthologous gene clusters, we reconstructed a maximum-likelihood (ML) phylogenetic tree to resolve the evolutionary relationships among the ten fungal strains. The phylogenetic reconstruction revealed a monophyletic clade comprising *C. fumosorosea* and *C. militaris*, distinct from the *C. javanica* lineage (Figure 3C; Supplementary Table S4), indicating a shared ancestry among these taxa relative to the out-group species. The evolutionary dynamics of the gene family expansion/contraction were reconstructed onto the phylogeny (Figure 3C), identifying 74 significantly expanded and 413 contracted gene families in *C. javanica* Bd01. To elucidate the functional implications of the lineage-specific expansions, we performed eukaryotic orthologous group (KOG) annotation on the gene families diverging at the *C. javanica* Bd01 speciation node. The KOG profiling (Figure 3D) revealed ABC transporters as the predominant functional category among the expanded families, surpassing even uncharacterized loci. The comparative CAZy annotation demonstrated a pronounced enrichment of glycoside hydrolases (GHs) in *C. javanica* Bd01 relative to the allied entomopathogens (Supplementary Table S5), particularly the GH18 chitinases and carbohydrate-binding module CBM50 domains.

### 2.3. Genome-Wide Identification and Characterization of GH18 Genes in Cordyceps javanica Bd01

Twenty-seven candidate GH18 genes were initially identified in the *C. javanica* Bd01 genome through homology-based screening using Hidden Markov Models (HMMs) integrated with the CAZy, InterPro, and SwissProt databases. The subsequent structural validation via the Conserved Domain Database (CDD) and the SMART platform confirmed the presence of conserved catalytic domains across all the sequences. Following stringent quality filtering to exclude truncated sequences (<100 amino acids) and low-identity homologs, the final set of 27 GH18 genes was retained for further analysis (Supplementary Table S6).

### 2.4. Conserved Motif and Chromosomal Location of GH18 Genes in Cordyceps javanica Bd01

**Figure 4:**
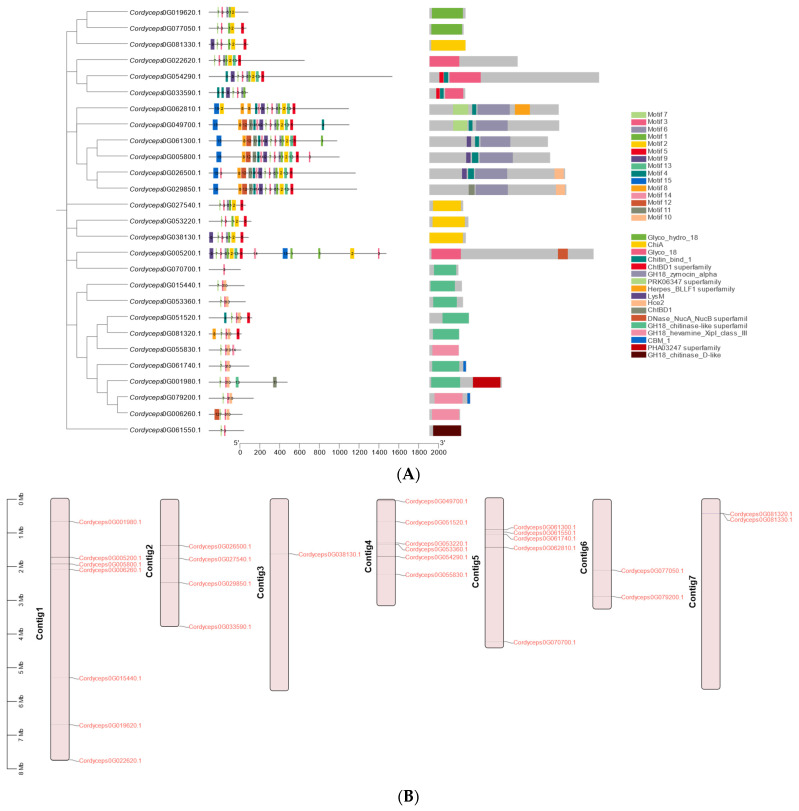
Conserved motif (**A**) and chromosomal location (**B**) of GH18 genes in *Cordyceps javanica* Bd01. Note: Scale bars denote length of amino acids. Motif color consistency is maintained across panels to emphasize structural–functional correlations, with the numbers representing motif numbering.

Conserved motif analysis, alongside subcellular localization predictions and transmembrane domain profiling, served as a critical tool for inferring the functional attributes of the GH18 proteins. Using the MEME suite, we identified 15 conserved motifs (designated motifs 1–15) across the GH18 family (Figure 4A). The core motifs, including motifs 1, 2, 3, 7, and 10, were ubiquitously detected in the majority of the GH18 proteins. Notably, *Cordyceps0G049700.1* harbored all 15 motifs, with motif 3 being universally conserved across all GH18 members, whereas *Cordyceps0G070700.1* retained only a single motif (Figure 4A). However, we noted a significant inter-clan divergence, suggesting that motif composition correlates with functional specialization within the GH18 subfamilies. This pattern implies conserved functional roles among the clan-specific members, with select motifs potentially driving lineage-dependent catalytic activities.

The chromosomal mapping of the 27 GH18 genes in *C. javanica* Bd01 demonstrated an uneven distribution across seven genomic contigs (Figure 4B). Contig 1 exhibited the highest density (seven genes), followed by contig 4 (six genes), while contig 3 contained a single GH18 locus. The spatial clustering analysis identified gene-rich regions predominantly localized to the proximal and medial segments of the contigs.

### 2.5. Phylogenetic Analysis of GH18 Family Proteins

**Figure 5:**
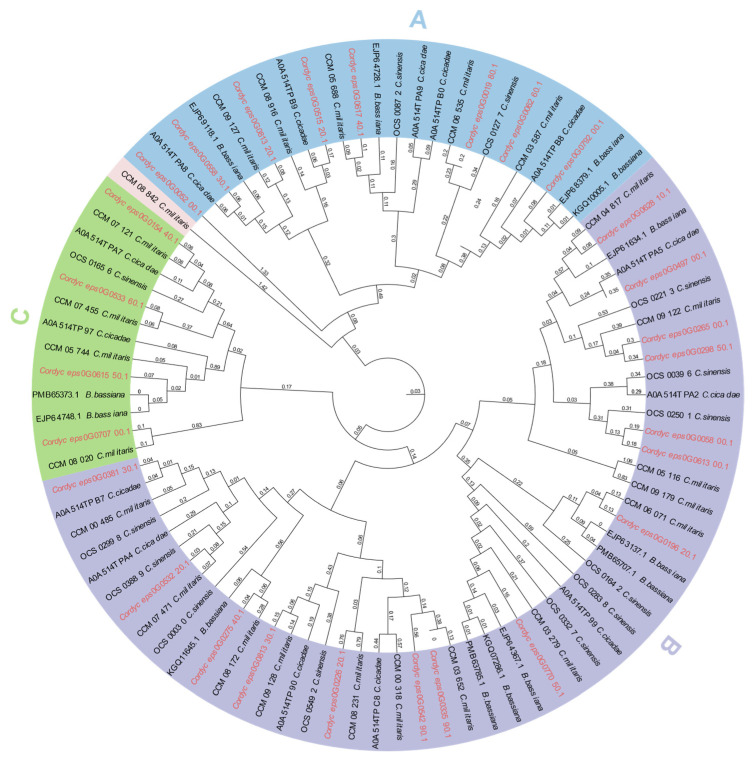
Phylogenetic relationship of GH18 from *Cordyceps javanica* Bd01 and four other fungal species. Note: Phylogenetic relationships were reconstructed via maximum-likelihood methodology using a curated multiple sequence alignment of GH18 catalytic domain amino acids. Nodal support was assessed through 1000 bootstrap replicates, with values ≥70% indicating significant topological confidence. *Cordyceps javanica* Bd01 GH18 genes are marked with red typeface.

To elucidate the functional divergence and evolutionary trajectories of the GH18 chitinases across taxa, we reconstructed a maximum-likelihood phylogenetic tree comprising the 27 *C. javanica* Bd01 GH18 protein sequences and 63 orthologs from four phylogenetically related fungal species: *C. sinensis*, *C. militaris*, *C. cicadae*, and *B. bassiana*. The resulting phylogeny resolved the *C. javanica* GH18 chitinases into three distinct clades (designated A, B, and C; Figure 5), a clustering pattern that mirrored the domain-based classification observed in the conserved structural analyses (Figure 4A).

### 2.6. Characterization of Physicochemical Properties

**Figure 6:**
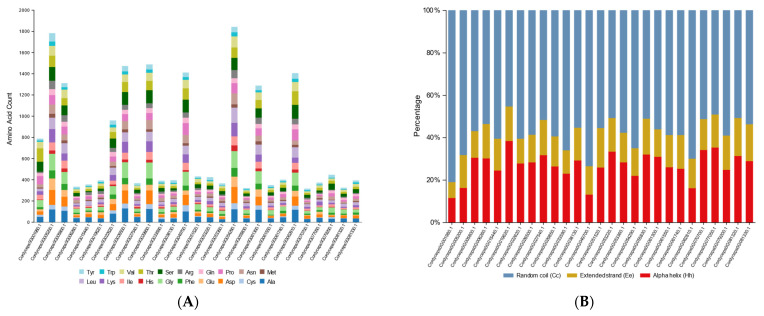
(**A**) Amino acid composition of GH18 genes from *Cordyceps javanica* Bd01; (**B**) percentage of secondary structure elements in *Cordyceps javanica* Bd01 GH18 genes.

The computational analysis using the ProtParam tool revealed substantial diversity in the physicochemical properties of the *C. javanica* Bd01 GH18 chitinases. These enzymes exhibited a broad variation in polypeptide length (315–1842 amino acids) and molecular mass (14.03–81.41 kDa), with theoretical isoelectric points (pI) spanning from acidic (4.40) to near-neutral (7.92) ranges (Table 2). The sequence analysis demonstrated a predominance of negatively charged residues (aspartate/glutamate) over positively charged counterparts (arginine/lysine) across all the chitinases, with the exception of *Cordyceps0G055830.1*, which displayed reversed polarity. The stability metrics indicated that most of the chitinases exhibited instability indices below 40, suggesting favorable in vivo half-life characteristics. The grand average of hydropathy (GRAVY) values were universally negative, consistent with hydrophilic protein architectures. The aliphatic indices ranged from 59.48 to 89.41, with a tripartite distribution: approximately 30% of the chitinases exceeded 75 (indicative of thermostable adaptations), 40% clustered between 65 and 75, and the remaining 30% averaged ~64. This marked heterogeneity implies divergent temperature optima and thermal stability profiles among the isoforms originating from a single organism. The amino acid composition profiling (Figure 6A) identified alanine and glycine as the most abundant residues in the *C. javanica* Bd01 chitinases. These enzymes were further characterized by elevated proportions of leucine, serine, threonine, aspartate, valine, proline, and isoleucine. The secondary structure predictions highlighted differential propensities for α-helix versus β-sheet formation across the isoforms, reflecting conformational diversity within the GH18 family.

### 2.7. Secondary and Tertiary Structure Analysis

The secondary structure analysis revealed a predominance of random coil conformations across all the chitinases, accounting for a mean proportion of 58.3% (Figure 6B). α-Helices and extended β-strands constituted the secondary structural components at 27% and 14.7%, respectively.

**Figure 7:**
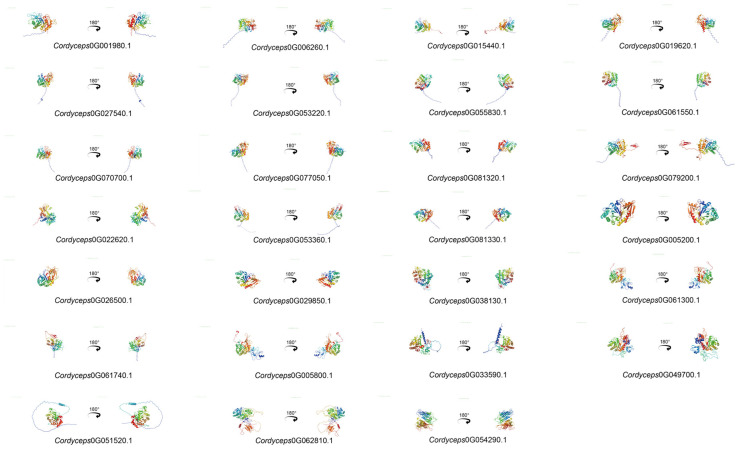
3D structure of *Cordyceps javanica* Bd01 GH18 genes. Note: the left and right plots represent symmetrical structures rotated 180° along the *y*-axis.

The tertiary architecture of proteins governs their functional specificity, including ligand binding, protein–protein/nucleotide interactions, and phenotypic manifestations of mutational variants. To resolve the three-dimensional (3D) conformations of the *C*. *javanica* Bd01 GH18 chitinases, homology modeling was performed using the SWISS-MODEL server, leveraging templates from homologous proteins with experimentally resolved crystal structures. The predicted 3D models demonstrated a high intra-subfamily structural conservation with a pronounced divergence observed across the distinct subfamilies (Figure 7).

### 2.8. cis-Regulatory Architecture of GH18 Chitinase Genes in Cordyceps javanica Bd01

**Figure 8:**
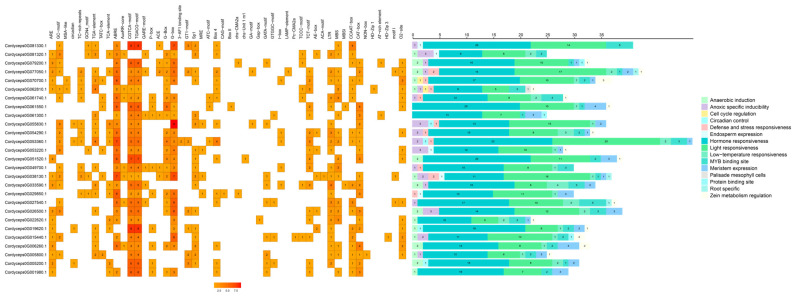
Diagram of cis-acting elements of the promoter sequence of the GH18 genes in *Cordyceps javanica* Bd01. Note: Motif Type Abundance (left matrix): Heatmap depicts the distribution of cis-regulatory element (CRE) types across GH18 promoters. Each row represents a CRE category (e.g., hormone-responsive elements, stress-inducible motifs), with columns corresponding to fungal species/gene clusters. Color intensity (orange gradient) correlates with motif copy number, scaled per row (darker hues = higher abundance). Sequence Occupancy (right stacked bars): Bar plots quantify cumulative nucleotide spans occupied by CREs per promoter. *x*-axis accounts for promoter length (−2000 bp to TSS).

The systematic analysis of the 2000 bp promoter regions upstream of the transcription start sites (TSSs) for all 27 GH18 chitinase genes in *C. javanica* Bd01 revealed a complex cis-regulatory landscape characterized by functional diversification and evolutionary constraint. The bioinformatic interrogation using PlantCARE identified hormone-responsive elements (HREs) as the most prevalent and phylogenetically conserved regulatory motif class, ubiquitously distributed across all the GH18 promoters (Figure 8). Notably, 90% of the promoters harbored the ABA-responsive *ABRE* (ACGT-containing motif), stress-inducible *G-BOX* (CACGTG), and jasmonate-responsive *CGTCA* elements, suggesting the coordinated regulation of chitinolytic activity under biotic/abiotic stress and host–pathogen interplay.

## 3. Discussion

The complete genome sequencing of *C. javanica* Bd01 revealed a total number of 78.1 million clean reads (~229.6× coverage), and the BUSCO analysis determined 98.28% completeness with a contig N50 length of 5.4 Mb. The resulting genome assembly was based on eight contigs with a 53.18% GC content, 9590 protein-coding genes, 43 rRNA genes, and 131 tRNA genes. The repetitive sequence was 0.34 Mb, which represented 1.04% of the total genome. These findings are in line with Yu et al. [33]; their genomic characterization of *Fusarium solani* KMZW-1 established a total assembly size of 47.24 Mb (47,239,278 bp) organized into 27 contigs, exhibiting a guanine-cytosine (GC) composition of 51.16%. The genome completeness was assessed as 97.93% through the BUSCO analysis. The DFVF sequence was assigned the accession number *Fusarium*0G092560.1, and AntiSMASH predicted 35 biosynthetic gene clusters (BGCs) linked to secondary metabolism. Notably, the genome of *C. cicadae* strain CCAD02 (the sexual morph of *I. cicadae*) was sequenced using an Illumina/Nanopore hybrid assembly with data generated from asexual fruiting body-derived DNA [34]. Both the genomes exhibited comparable sizes, ranging between 33.8 and 33.9 Mb.

Tracing gene family dynamics (expansion/contraction) across phylogenetically related lineages enables the systematic identification of molecular drivers governing species-specific adaptation and evolutionary divergence [35]. By the comparative genomic analysis, we found that the chitinases (GH18) were significantly expanded in genes in the genome of *C. javanica* Bd01. The GH18 chitinases represent evolutionarily conserved multigene families ubiquitously distributed across taxa, spanning prokaryotes to eukaryotes. These enzymes mediate critical physiological functions, including cellular growth and development, substrate hydrolysis for nutrient acquisition, host–microbe interactions, and pathogenicity–defense dynamics, underscoring their indispensable role in organismal adaptation and evolutionary success across ecological niches [36,37]. Notably, chitinases derived from *B. bassiana* and related entomopathogenic fungi have been characterized as critical virulence determinants, directly mediating fungal pathogenicity against arthropod and nematode hosts during infective processes [38]. Chitinolytic fungi frequently utilize chitin-rich substrates or organisms as nutritional sources. The subgroup B chitinases exhibit dual functionality in fungal physiology, serving roles in both nutrient acquisition and host invasion/pathogenesis [24]. In mycoparasitic and entomopathogenic fungi, these enzymes are typically inducible under nutrient-limiting conditions, particularly in the presence of chitin or host-derived carbon sources. For instance, the transcriptional up-regulation of key subgroup B chitinases—including *Chit33* in *Trichoderma harzianum*, *Ech30* in *T. atroviride*, and *Chi2* in *Metarhizium anisopliae*—is triggered during starvation but suppressed by readily metabolizable substrates such as glucose [24]. Functional validation studies further underscore their critical role in insect pathogenesis. The targeted disruption of *Chi2* in *M. anisopliae* significantly attenuated fungal virulence toward insect hosts, whereas transgenic overexpression strains demonstrated enhanced host mortality rates, confirming a dose-dependent relationship between chitinase activity and pathogenic efficacy [28]. The other two subgroups (subgroups A and C) of fungal chitinases are also related to pathogenesis [39,40,41,42]. The subcellular compartmentalization and localization of proteins, including enzymatic components, is intrinsically associated with their biological functions and catalytic specificity. In the *C. cicadae* genome, Lu et al. [34] documented 135 carbohydrate-active enzymes (CAZymes), including 16 GH18 family members, a finding consistent with comparative genomic analyses demonstrating enriched GH18 chitinase inventories in entomopathogenic fungi relative to their phytopathogenic and mammalian-pathogenic counterparts [43]. Our annotation identified 27 GH18 chitinase genes, including several candidates with putative pathogenicity determinants. The systematic characterization of their subcellular localization patterns may elucidate enzymatic pathway specialization and inform rational experimental design in functional studies.

To mechanistically characterize the *C. javanica* Bd01 chitinase, we implemented an integrated computational workflow incorporating the following: physicochemical profiling (molecular mass, theoretical pI, instability index), multilevel structural characterization (primary sequence motifs, secondary structure elements, tertiary fold prediction), functional domain architecture (Pfam, SMART, CDD databases), and homology modeling (SWISS-MODEL, Phyre2). However, the function of GH18 in the pathogenesis of *C. javanica* Bd01 is unknown and needs to be further investigated, and we are only making predictions based on the existing conditions. Future research utilizing RNA-seq under host cuticle induction will help in quantifying the expression dynamics of the expanded GH18 genes.

The molecular weight (MW) of the chitinolytic enzymes serves as a determinant factor in selecting chromatographic strategies. The observed MW range (14.03–81.41 kDa) guided the implementation of gel-filtration chromatography with Sephadex G-100 SF (fractionation range: 40–120 kDa), optimized through empirical calibration to resolve the target chitinolytic enzymes. The comparative orthology assessment demonstrated 1:1 orthologous relationships between the *C. javanica* Bd01 GH18 genes and the corresponding loci in four phylogenetically representative species, indicative of a phylogenetically conserved GH18 evolution through vertical inheritance from ancestral GH18 loci. The computational profiling of the *Cordyceps javanica* Bd01 GH18 chitinases revealed significant electrostatic heterogeneity across the isoforms, characterized by variable ratios of basic (e.g., arginine, lysine) to acidic (e.g., aspartate, glutamate) residues. This physicochemical diversity necessitates customized isoelectric focusing protocols to accommodate pH-dependent solubility profiles during protein purification. Notably, 79% (21/27) of the GH18 chitinases exhibited a predominantly acidic residue composition (pI range: 4.40–5.80), consistent with their predicted extracellular localization and functional specialization in cuticle degradation [44,45]. This charge distribution informed our implementation of tandem anion-exchange chromatography, sequentially employing weak (DEAE-Sepharose) and strong (Q-Sepharose) exchangers—a strategy consistent with the successful purification protocols for *B. bassiana* chitinases using DEAE-cellulose and Mono Q matrices [30,46]. The emerging evidence underscores the regulatory role of the nascent polypeptide charge states in modulating polysome-associated biosynthesis [47], suggesting that the acidic pI values observed here may facilitate ribosomal processivity during chitinase translation. The stability predictions based on the instability indices revealed a dichotomy: proteins with sub-40 indices exhibited predicted in vivo half-lives exceeding 16 h (n = 17), while those exceeding 40 demonstrated <5 h persistence [48]. Notably, 10 *C. javanica* Bd01 chitinases displayed atypical instability profiles, which is potentially indicative of evolutionarily recent gene duplication events given the strain’s complement of thermodynamically stable ancestral paralogs [49]. These computational insights align with the enhanced kinetic stability required for entomopathogenic enzymatic function and industrial biocatalysis [50]. The secondary structure analysis demonstrated conserved folding principles: alanine (12.4%), leucine (9.7%), aspartate (8.3%), and proline (6.1%) exhibited a pronounced α-helix propensity, whereas valine (10.2%), isoleucine (7.9%), threonine (6.7%), glycine (5.8%), and serine (5.4%) favored the β-sheet conformation [49]. The observed structural dominance of α-helices (38.2 ± 3.1%) and random coils (45.6 ± 4.3%) across the chitinase repertoire suggests an evolutionary optimization balancing conformational flexibility with catalytic domain rigidity [51].

To characterize the *C. javanica* Bd01 GH18 gene family, 27 representative loci were successfully identified and structurally profiled. The secondary structure prediction revealed all the GH18 chitinases exhibit conserved α-helix (18.3 ± 2.1%), β-sheet (12.7 ± 1.8%), and random coil (69.0 ± 3.4%) architectures, with coil domains predominating across all the isoforms. This structural plasticity suggests the potential involvement of alternative splicing mechanisms in generating chitinase diversity, enabling the differential expression of isoform-specific catalytic modules during distinct infection phases or host interactions—a putative evolutionary strategy for expanding host tropism.

The functional characterization of the core promoter elements in filamentous fungi has primarily focused on the TATA box, CCAAT motifs, and CT-rich regions. Regulatory elements ubiquitous in higher eukaryotes—including Initiator (Inr), Downstream Promoter Element (DPE), Motif Ten Element (MTE), TFIIB Recognition Element (BRE), and CpG islands—remain uncharacterized in these systems. Notably, promoter engineering studies in *Aspergillus* spp. have demonstrated enhanced transcriptional activity through the multiplexed integration of cis-acting regulatory modules [52]. The Initiator (Inr) element, spanning the transcription start site (TSS) region, enhances promoter activity through interactions with the TFIID subunits TAF1/TAF2, as demonstrated in the *gpdA* promoter of *Aspergillus niger* [53]. Abscisic acid (ABA)-responsive elements coordinate osmotic and oxidative stress responses by recruiting basic leucine zipper (bZIP) transcription factors such as Atf1, which activate pathogenesis-related genes in *Magnaporthe oryzae* [54]. Jasmonate-responsive elements facilitate defense signaling mimicry during host–pathogen interactions, regulating secondary metabolite biosynthetic clusters, exemplified by the *TRI5* promoter in *F. graminearum* [55]. Heat shock elements (HSEs) bind heat shock factor (HSF) to modulate the expression of heat shock protein (HSP) genes, notably *HSP104* in *Saccharomyces cerevisiae* [56]. Light-responsive elements govern conidiation and circadian rhythm-associated genes, including the *blr-1/2* photoreceptor loci in *T. atroviride* [57]. G-boxes are a class of cis-acting elements, including the ACGT family, that are capable of responding to a variety of environmental cues, including ABA, light, UV, injury, and pathogen signaling. Jasmonate signaling pathways, often hijacked by pathogens to suppress plant/insect immune responses, may drive GH18 expression during cuticle penetration. ABA-mediated osmotic stress adaptation could enhance fungal survival under host-derived oxidative bursts [58,59]. This cis-regulatory blueprint provides mechanistic insights into shared TF-binding motifs (e.g., *ABRE*) with plant/insect systems and may reflect molecular mimicry strategies to subvert host defenses. Modular promoter elements (e.g., stress-inducible *G-BOX*) could be harnessed to design environmentally responsive chitinase expression systems for precision biocontrol applications. These findings establish *C. javanica* GH18 promoters as a model system for studying fungal transcriptional adaptation and informing synthetic biology approaches to enhance entomopathogen efficacy. Future functional studies should prioritize the in planta validation of ABA/jasmonate crosstalk in regulating GH18-mediated virulence.

In summary, 27 GH18 chitinase genes exhibiting structural heterogeneity (intron-exon architecture), polypeptide length variation (14.03–81.41 kDa), and divergent physicochemical profiles were systematically annotated in *C. javanica* Bd01. The observed negative GRAVY indices (grand average of hydropathicity) and elevated aliphatic indices collectively corroborate the enzymes’ inherent hydrophilicity and thermodynamic robustness. The integrative phylogenomic–structural analyses revealed a striking evolutionary conservation across these chitinases, suggesting the preservation of a shared catalytic architecture governing chitinolytic function. This computational framework provides the following:

- Actionable biochemical parameters for targeted in vitro purification and functional characterization;
- Structural blueprints facilitating the homology-based functional prediction of uncharacterized GH18 paralogs;
- The evolutionary rationale for biotechnological exploitation, particularly in developing thermostable chitinolytic cocktails for industrial biocatalysis and biocontrol formulations.

## 4. Materials and Methods

### 4.1. Culture of Cordyceps javanica Bd01

The strain *C. javanica* Bd01 was isolated from the dead larvae of *Xylotrechus quadripes.* The fungal isolate belonged to the collection of the Institute of Microbiology, Chinese Academy of Sciences, Conservation No. CGMCC23078. The fungal isolate underwent morpho-taxonomic characterization complemented by molecular identification via sequencing of the ITS1-5.8S-ITS4 rRNA cluster. The annotated internal transcribed spacer (ITS) sequence of *C*. *javanica* Bd01 has been archived in the NCBI GenBank repository under accession number MZ831846 [60]. For routine maintenance, the isolate was cultured on Sabouraud dextrose agar supplemented with 1% (*w*/*v*) yeast extract (SDAY medium) [2]. Standardized axenic cultivation involved incubating Bd01 on SDAY at 27 ± 0.5 °C with 70% ± 5% relative humidity under a 12 h/12 h dark/light photoperiod, achieving optimal hyphal extension rates over a 14-day cultivation period.

### 4.2. DNA Extraction and Sequencing

Genomic DNA was isolated from *C*. *javanica* Bd01 axenic cultures grown on SDAY medium using the Omega Fungal DNA Kit (D3390-02, Omega Bio-tek, Illumina, San Diego, CA, USA), with purity (OD_260/280_ = 1.8–2.0) and concentration (>15 μg) verified via TBS-380 fluorometric quantification (Turner BioSystems Inc., Sunnyvale, CA, USA).

A dual-platform strategy combining PacBio SMRT (Single Molecule Real-Time) and Illumina sequencing was implemented:

Illumina Library Preparation

Fragmentation: 5 μg DNA sheared to 400–500 bp using Covaris M220 (peak incident power: 50 W, duty factor: 20%, 200 cycles/burst). End Repair/A-Tailing: 5′ phosphorylation and 3′ dA-tailing performed with NEXTflex™ Rapid DNA-Seq reagents (Biomarker Technologies Co., Ltd., Beijing, China).

Adapter Ligation: T4 DNA ligase-mediated adapter hybridization (Illumina TruSeq indexes).

PCR Enrichment: 8-cycle amplification with KAPA HiFi HotStart ReadyMix (KAPA Biosystems, Wilmington, MA USA).

Sequencing: 2 × 150 bp paired-end reads generated on HiSeq X Ten (Illumina, San Diego, CA, USA).

PacBio SMRTbell Library Construction

Size Selection: 8 μg DNA sheared via g-TUBE (Covaris, MA, USA) centrifugation (6000× *g*, 60 s; Eppendorf 5424, Eppendorf, NY, USA).

Damage Repair: end-repair/purification using SMRTbell Template Prep Kit 1.0 (Pacific Biosciences, Menlo Park, CA, USA).

Adapter Ligation: SMRTbell adapters attached via T4 ligase (Pacific Biosciences, Menlo Park, CA, USA).

Bead-Based Cleanup: triple purification with 0.45× AMPure XP beads (Beckman Coulter Genomics, Danvers, MA, USA).

Sequencing: 10 kb insert library sequenced on Sequel II System (v9.0 chemistry).

Quality Assurance

ONT protocols were rigorously followed for:

Pre-library QC (Qubit 4.0, Agilent 4200 TapeStation, Oxford, UK).

Post-library validation (Fragment Analyzer, qPCR, Oxford, UK).

### 4.3. Genome Assembly and Annotation

The genomic assembly was reconstructed through hybrid integration of PacBio long-read and Illumina short-read sequencing data. Primary signal output from both platforms was processed through base-calling algorithms, converting raw electrical/optical signals into nucleotide sequences. These raw reads, comprising standardized read identifiers, nucleotide strings, and Phred-scaled quality scores, were archived in FASTQ format for downstream computational workflows. Raw sequencing reads underwent quality control processing, including adaptive trimming of low-quality bases (Q-score < 20) and removal of adapter sequences using Trimmomatic v0.39. High-fidelity reads were subsequently assembled into contigs via CANU v2.1.1 with default parameters. To enhance assembly accuracy, PacBio long-read contigs were error-corrected using Illumina short reads through Pilon v1.24 with iterative polishing (3 cycles).

The predicted proteins were analyzed via BLAST (e-value: 1 × 10^−5^, https://blast.ncbi.nlm.nih.gov/Blast.cgi, (accessed on 22 October 2024)) against Nr [61], Swiss-Prot [62], TrEMBL [62], KEGG [63], and KOG [64]. Blast2go [65] was used for GO [66] annotation. Hmmer [67] was used for Pfam [68] annotation.

### 4.4. Orthologous and Phylogenomic Analysis

We used OrthoFinder version 2.5.5 [69] to analyze gene families of ten entomopathogens, including *C. javanica* Bd01, *C. javanica* IJ1, *C. javanica* IJ2, *Cordyceps fumosorosea* ARSEF2679, *Cordyceps militaris* CM01, *C. militaris* ATCC34164, *Akanthomyces lecanii* RCEF1005, *B*. *bassiana* ARSEF2860, *Moelleriella libera* RCEF2490, and *Metarhizium anisopliae* JEF290. Phylogenomic analysis of 4096 single-copy orthologous gene families was conducted across six strains using MUSCLE v5.0 for multiple sequence alignment (MSA) [70]. Orthologous sequences were concatenated into a super matrix via a custom Perl script. Following alignment refinement with Gblocks v0.91b (parameters: minimum block length = 10, gap positions allowed in final blocks) [71], a maximum-likelihood phylogeny was reconstructed in MEGA v11.0.13 under the Jones–Taylor–Thornton (JTT) substitution model with gamma-distributed rate variation (α = 1.0) and 1000 bootstrap replicates. Topological robustness was assessed through partial gap deletion (95% site coverage threshold) and invariant site exclusion [72].

### 4.5. Identification and Characterization of GH18 Gene Family in Cordyceps javanica Bd01

Protein sequences annotated in *C*. *javanica* Bd01 were subjected to homology-based functional profiling through a Hidden Markov Model (HMM)-guided search against the Swiss-Prot (UniProtKB/Swiss-Prot release 2024_03) [62], InterPro (v97.0) [73], and CAZy (v11.0) [74] databases using HMMER v3.3.2 with an e-value cutoff of 1 × 10^−5^. Conserved domain validation for GH18 family members was performed via iterative sequence analysis using SMART (v9.0) [75] and the NCBI Conserved Domain Database (CDD v3.20) [76]. Physicochemical profiling of GH18 chitinases—including amino acid composition, molecular weight (MW), instability index (II), aliphatic index (AI), grand average of hydropathicity (GRAVY), and isoelectric point (pI)—was computationally derived using the ExPASy ProtParam algorithm (https://web.expasy.org/protparam/ (accessed on 25 October 2024)) [77]. Subcellular localization predictions were generated through WoLF PSORT (v1.0; https://wolfpsort.hgc.jp/ (accessed on 26 October 2024)) [78], integrating amino acid sequence features with eukaryotic sorting signal patterns.

### 4.6. Conserved Domain, Conserved Motif Analyses, and Chromosomal Location

Conserved domain architectures were systematically profiled through comparative analysis against the NCBI Conserved Domain Database (CDD v3.20) using the Batch CD-Search tool (e-value cutoff: 0.01, max hits: 500) [76]. Putative conserved motifs within GH18 chitinases were computationally identified via MEME Suite v5.5.7 (default parameters: maximum motifs = 15, site distribution = any number of repetitions) [79]. Secondary structures (α-helices, β-sheets, random coils) were predicted using SOPMA v2.16.0 (window width = 17, similarity threshold = 8) [80], while tertiary structure homology modeling was performed through the SWISS-MODEL workspace v1.0.2 (global model quality estimation GMQE > 0.7) [81].

## Figures and Tables

**Table 1:** Genome features of *Cordyceps javanica* Bd01.

Sample	*Cordyceps javanica* Bd01
Coverage (fold)	229.59X
No. of all sequences	698,137
Bases in all sequences (bp)	7,808,156,751
Longest length (bp)	193,316
N50 length (bp)	18,161
N90 length (bp)	4902
G+C content	53.18
No. of all contigs	8
Contig Length (bp)	34,008,118
No. of large contigs (>1000 bp)	8
Bases in large contigs (bp)	34,008,118
Contig N50 (bp)	5,679,179
Contig N90 (bp)	3,285,737
Gene number	9590
Gene total length (bp)	17,091,976
Gene average length (bp)	1782.27
ExonLen (bp)	15,026,841
AveExonLen (bp)	568.12
ExonNum	26,450
AveExonNum	2.76
rRNA	43
tRNA	131

**Table 2:** Physicochemical properties of the GH18 gene family in *Cordyceps javanica* Bd01.

Gene Name	Number of Amino Acids	Molecular Weight (kDa)	Total Number of Negatively Charged Residues (Asp + Glu)	Total Number of Positively Charged Residues (Arg + Lys)	Theoretical pI	Instability Index	Aliphatic Index	GRAVY	Subcellular Localization
*Cordyceps0G001980.1*	787	81.41	49	47	7.92	39.67	63.52	−0.206	extr
*Cordyceps0G005200.1*	1782	19.91	249	206	5.33	40.35	62.25	−0.604	nucl
*Cordyceps0G005800.1*	1310	14.35	157	126	5.55	44.52	76.86	−0.268	plas
*Cordyceps0G006260.1*	333	35.07	29	17	4.52	33.17	68.68	−0.186	extr
*Cordyceps0G015440.1*	353	38.95	50	31	4.73	43.44	89.41	−0.194	mito
*Cordyceps0G019620.1*	392	43.82	57	43	5.03	48.63	78.65	−0.329	mito
*Cordyceps0G022620.1*	958	10.54	107	92	5.58	45.09	64.73	−0.421	extr
*Cordyceps0G026500.1*	1473	15.9	180	148	5.39	28.36	63.65	−0.443	extr
*Cordyceps0G027540.1*	367	39.06	33	28	6.11	46.74	83.32	−0.126	extr
*Cordyceps0G029850.1*	1486	16.06	182	143	5.2	30.46	67.27	−0.412	extr
*Cordyceps0G033590.1*	389	42.6	36	32	6.2	33.37	64.73	−0.456	extr
*Cordyceps0G038130.1*	395	44.06	54	33	4.72	37.83	67.72	−0.508	extr
*Cordyceps0G049700.1*	1409	15.1	138	125	5.83	43.01	65.44	−0.376	extr
*Cordyceps0G051520.1*	430	45.22	45	36	5.44	30.03	73.35	−0.231	extr
*Cordyceps0G053220.1*	423	46.19	39	38	6.53	26.44	73.22	−0.319	extr
*Cordyceps0G053360.1*	365	39.84	31	25	5.74	38.65	80.47	−0.147	extr
*Cordyceps0G054290.1*	1842	20.43	251	204	5.63	36.42	72.49	−0.461	extr
*Cordyceps0G055830.1*	320	34.08	29	30	7.53	24.01	76.34	−0.218	extr
*Cordyceps0G061300.1*	1286	14.03	150	127	5.68	43.02	73.79	−0.265	extr
*Cordyceps0G061550.1*	348	36.71	24	23	6.12	31.95	84.45	0.009	extr
*Cordyceps0G061740.1*	401	42.25	28	24	4.94	36.66	59.48	−0.183	extr
*Cordyceps0G062810.1*	1404	15.14	140	104	5.3	41.7	70.89	−0.236	mito
*Cordyceps0G070700.1*	315	34.54	35	32	5.69	28.53	78.44	−0.320	extr
*Cordyceps0G077050.1*	372	41.41	52	47	5.63	33.53	64.87	−0.483	extr
*Cordyceps0G079200.1*	445	46.94	24	23	6.29	41.27	61.21	−0.290	extr
*Cordyceps0G081320.1*	324	33.98	31	19	4.4	37.51	80.46	−0.108	extr
*Cordyceps0G081330.1*	392	43.13	34	31	5.5	35.96	73.67	−0.246	extr

Note: GRAVY (grand average of hydropathy): hydrophobicity score (Kyte–Doolittle scale); negative values denote hydrophilic character. Subcellular localization indicates the predicted location, where extr indicates extracellular, nucl indicates nucleus, plas indicates plasma membrane, and mito indicates mitochondria.

## Data Availability

The original contributions presented in this study are included in the article and Supplementary Materials.

## Supplementary Materials

The following supporting information can be downloaded at: https://www.mdpi.com/article/10.3390/ijms26052031/s1.

## References

[1] Luangsa-Ard J.J., Hywel-Jones N.L., Manoch L., Samson R.A. (2005). On the Relationships of *Paecilomyces* Sect. Isarioidea Species1. Mycol. Res..

[2] Gallou A., Serna-Domínguez M.G., Berlanga-Padilla A.M., Ayala-Zermeño M.A., Mellín-Rosas M.A., Montesinos-Matías R., Arredondo-Bernal H.C. (2016). Species Clarification of Isaria Isolates Used as Biocontrol Agents against Diaphorina Citri (Hemiptera: Liviidae) in Mexico. Fungal Biol..

[3] Xie L., Han J.H., Kim J.J., Lee S.Y. (2016). Effects of Culture Conditions on Conidial Production of the Sweet Potato Whitefly Pathogenic Fungus *Isaria Javanica*. Mycoscience.

[4] Pan H.R., Lin Y., Lv B.K. (2019). Evaluation of Isaria javanica WH-EP-1 for the control of *Bemisia argentifolii* biotype B. Compendium of Research on Agricultural Improvement in Hualien District.

[5] Hasan W.A., Assaf L.H., Abdullah S.K. (2012). Occurrence of Entomopathogenic and Other Opportunistic Fungi in Soil Collected from Insect Hibernation Sites and Evaluation of Their Entomopathogenic Potential. Bull. Iraq Nat. Hist. Mus..

[6] Min C. (2014). Wettable Powder Development of Isaria Javanica for Control of the Lesser Green Leafhopper, Empoasca Vitis. J. Biol. Control.

[7] Park S.E., Kim J.C., Lee S.J., Lee M.R., Kim S., Li D., Baek S., Han J.H., Kim J.J., Koo K.B. (2018). Solid Cultures of Thrips-Pathogenic Fungi *Isaria Javanica* Strains for Enhanced Conidial Productivity and Thermotolerance. J. Asia-Pac. Entomol..

[8] Hu Q., Liu S., Yin F., Cai S., Zhong G., Ren S. (2011). Diversity and Virulence of Soil-Dwelling Fungi *Isaria* Spp. and *Paecilomyces* Spp. against *Solenopsis invicta* (Hymenoptera: Formicidae). Biocontrol Sci. Technol..

[9] Lopes R.S., Svedese V.M., Portela A.P.A.S., Albuquerque A.C., Luna-Alves Lima E.A. (2011). Virulência e Aspectos Biológicos de Isaria Javanica (Frieder & Bally) Samson & Hywell-Jones Sobre Coptotermes Gestroi (Wasmann) (Isoptera: Rhinotermitidae). Arq. Inst. Biol..

[10] Cabanillas H.E., Jones W.A. (2009). Effects of Temperature and Culture Media on Vegetative Growth of an Entomopathogenic Fungus Isaria Sp. (Hypocreales: Clavicipitaceae) Naturally Affecting the Whitefly, Bemisia Tabaci in Texas. Mycopathologia.

[11] Shimazu M., Takatsuka J. (2010). Isaria Javanica (Anamorphic Cordycipitaceae) Isolated from Gypsy Moth Larvae, Lymantria Dispar (Lepidoptera: Lymantriidae), in Japan. Appl. Entomol. Zool..

[12] Ortiz-Urquiza A., Keyhani N.O. (2013). Action on the Surface: Entomopathogenic Fungi versus the Insect Cuticle. Insects.

[13] Huang Z., Hao Y., Gao T., Huang Y., Ren S., Keyhani N.O. (2016). The Ifchit1 Chitinase Gene Acts as a Critical Virulence Factor in the Insect Pathogenic Fungus Isaria Fumosorosea. Appl. Microbiol. Biotechnol..

[14] Ortiz-Urquiza A., Keyhani N.O. (2016). Molecular Genetics of Beauveria Bassiana Infection of Insects. Adv. Genet..

[15] Valero-Jiménez C.A., Wiegers H., Zwaan B.J., Koenraadt C.J.M., van Kan J.A.L. (2016). Genes Involved in Virulence of the Entomopathogenic Fungus Beauveria Bassiana. J. Invertebr. Pathol..

[16] Wang C., Wang S. (2017). Insect Pathogenic Fungi: Genomics, Molecular Interactions, and Genetic Improvements. Annu. Rev. Entomol..

[17] Elad Y., Chet I., Henis Y. (1982). Degradation of Plant Pathogenic Fungi by Trichoderma Harzianum. Can. J. Microbiol..

[18] Inbar J., Chet I. (1995). The Role of Recognition in the Induction of Specific Chitinases during Mycoparasitism by Trichoderma Harzianum. Microbiology.

[19] Adams D.J. (2004). Fungal Cell Wall Chitinases and Glucanases. Microbiology.

[20] Gonfa T.G., Negessa A.K., Bulto A.O. (2023). Isolation, Screening, and Identification of Chitinase-Producing Bacterial Strains from Riverbank Soils at Ambo, Western Ethiopia. Heliyon.

[21] Henrissat B., Bairoch A. (1993). New Families in the Classification of Glycosyl Hydrolases Based on Amino Acid Sequence Similarities. Biochem. J..

[22] Kawase T., Saito A., Sato T., Kanai R., Fujii T., Nikaidou N., Miyashita K., Watanabe T. (2004). Distribution and Phylogenetic Analysis of Family 19 Chitinases in Actinobacteria. Appl. Environ. Microbiol..

[23] Seidl V. (2008). Chitinases of Filamentous Fungi: A Large Group of Diverse Proteins with Multiple Physiological Functions. Fungal Biol. Rev..

[24] Hartl L., Zach S., Seidl-Seiboth V. (2012). Fungal Chitinases: Diversity, Mechanistic Properties and Biotechnological Potential. Appl. Microbiol. Biotechnol..

[25] Adrangi S., Faramarzi M.A. (2013). From Bacteria to Human: A Journey into the World of Chitinases. Biotechnol. Adv..

[26] Stals I., Samyn B., Sergeant K., White T., Hoorelbeke K., Coorevits A., Devreese B., Claeyssens M., Piens K. (2010). Identification of a Gene Coding for a Deglycosylating Enzyme in Hypocrea Jecorina. FEMS Microbiol. Lett..

[27] Tzelepis G., Hosomi A., Hossain T.J., Hirayama H., Dubey M., Jensen D.F., Suzuki T., Karlsson M. (2014). Endo-β-N-Acetylglucosamidases (ENGases) in the Fungus Trichoderma Atroviride: Possible Involvement of the Filamentous Fungi-Specific Cytosolic ENGase in the ERAD Process. Biochem. Biophys. Res. Commun..

[28] Boldo J.T., Junges A., do Amaral K.B., Staats C.C., Vainstein M.H., Schrank A. (2009). Endochitinase CHI2 of the Biocontrol Fungus Metarhizium Anisopliae Affects Its Virulence toward the Cotton Stainer Bug Dysdercus Peruvianus. Curr. Genet..

[29] Staats C.C., Kmetzsch L., Lubeck I., Junges A., Vainstein M.H., Schrank A. (2013). Metarhizium Anisopliae Chitinase CHIT30 Is Involved in Heat-Shock Stress and Contributes to Virulence against Dysdercus Peruvianus. Fungal Biol..

[30] Fang W., Leng B., Xiao Y., Jin K., Ma J., Fan Y., Feng J., Yang X., Zhang Y., Pei Y. (2005). Cloning of Beauveria Bassiana Chitinase Gene Bbchit1 and Its Application to Improve Fungal Strain Virulence. Appl. Environ. Microbiol..

[31] Meng H., Wang Z., Meng X., Xie L., Huang B. (2015). Cloning and Expression Analysis of the Chitinase Gene Ifu-Chit2 from Isaria Fumosorosea. Genet. Mol. Biol..

[32] Wang C., Gao T., Huang Y., Huang Z. (2017). Effect of Ifchit1 Gene of Isaria Fumosorosea on Mortality, Oviposition and Oxidase Activities of Bemisia Tabaci. Biocontrol Sci. Technol..

[33] Yu J., Hussain M., Wu M., Shi C., Li S., Ji Y., Hussain S., Qin D., Xiao C., Wu G. (2024). Whole-Genome Sequencing of the Entomopathogenic Fungus Fusarium Solani KMZW-1 and Its Efficacy against Bactrocera Dorsalis. Curr. Issues Mol. Biol..

[34] Lu Y., Luo F., Cen K., Xiao G., Yin Y., Li C., Li Z., Zhan S., Zhang H., Wang C. (2017). Omics Data Reveal the Unusual Asexual-Fruiting Nature and Secondary Metabolic Potentials of the Medicinal Fungus Cordyceps Cicadae. BMC Genom..

[35] Mitreva M., Jasmer D.P., Zarlenga D.S., Wang Z., Abubucker S., Martin J., Taylor C.M., Yin Y., Fulton L., Minx P. (2011). The Draft Genome of the Parasitic Nematode Trichinella Spiralis. Nat. Genet..

[36] Gruber S., Seidl-Seiboth V. (2012). Self versus Non-Self: Fungal Cell Wall Degradation in Trichoderma. Microbiology.

[37] Nagpure A., Choudhary B., Gupta R.K. (2014). Chitinases: In Agriculture and Human Healthcare. Crit. Rev. Biotechnol..

[38] Berini F., Katz C., Gruzdev N., Casartelli M., Tettamanti G., Marinelli F. (2018). Microbial and Viral Chitinases: Attractive Biopesticides for Integrated Pest Management. Biotechnol. Adv..

[39] Carsolio C., Benhamou N., Haran S., Cortés C., Gutiérrez A., Chet I., Herrera-Estrella A. (1999). Role of the Trichoderma Harzianum Endochitinase Gene, Ech42, in Mycoparasitism. Appl. Environ. Microbiol..

[40] Woo S.L., Donzelli B., Scala F., Mach R., Harman G.E., Kubicek C.P., Del Sorbo G., Lorito M. (1999). Disruption of the Ech42 (Endochitinase-Encoding) Gene Affects Biocontrol Activity in Trichoderma Harzianum P1. Mol. Plant-Microbe Interact..

[41] Gruber S., Vaaje-Kolstad G., Matarese F., López-Mondéjar R., Kubicek C.P., Seidl-Seiboth V. (2011). Analysis of Subgroup C of Fungal Chitinases Containing Chitin-Binding and LysM Modules in the Mycoparasite Trichoderma Atroviride. Glycobiology.

[42] Gruber S., Kubicek C.P., Seidl-Seiboth V. (2011). Differential Regulation of Orthologous Chitinase Genes in Mycoparasitic Trichoderma Species. Appl. Environ. Microbiol..

[43] Shang Y., Xiao G., Zheng P., Cen K., Zhan S., Wang C. (2016). Divergent and Convergent Evolution of Fungal Pathogenicity. Genome Biol. Evol..

[44] Seidl V., Huemer B., Seiboth B., Kubicek C.P. (2005). A Complete Survey of Trichoderma Chitinases Reveals Three Distinct Subgroups of Family 18 Chitinases. FEBS J..

[45] Junges Â., Boldo J.T., Souza B.K., Guedes R.L.M., Sbaraini N., Kmetzsch L., Thompson C.E., Staats C.C., de Almeida L.G.P., de Vasconcelos A.T.R. (2014). Genomic Analyses and Transcriptional Profiles of the Glycoside Hydrolase Family 18 Genes of the Entomopathogenic Fungus Metarhizium Anisopliae. PLoS ONE.

[46] Havukkala I., Mitamura C., Hara S., Hirayae K., Nishizawa Y., Hibi T. (1993). Induction and Purification of *Beauveria Bassiana* Chitinolytic Enzymes. J. Invertebr. Pathol..

[47] Requião R.D., Fernandes L., de Souza H.J.A., Rossetto S., Domitrovic T., Palhano F.L. (2017). Protein Charge Distribution in Proteomes and Its Impact on Translation. PLoS Comput. Biol..

[48] Gasteiger E., Hoogland C., Gattiker A., Duvaud S., Wilkins M.R., Appel R.D., Bairoch A., Walker J.M. (2005). Protein Identification and Analysis Tools on the ExPASy Server. The Proteomics Protocols Handbook.

[49] Idicula-Thomas S., Balaji P.V. (2005). Understanding the Relationship between the Primary Structure of Proteins and Their Amyloidogenic Propensity: Clues from Inclusion Body Formation. Protein Eng. Des. Sel..

[50] Gohel V., Naseby D.C. (2007). Thermalstabilization of Chitinolytic Enzymes of *Pantoea Dispersa*. Biochem. Eng. J..

[51] Gouripur G.C., Kaliwal R.B., Kaliwal B.B. (2016). In Silico Characterization of Beta-Galactosidase Using Computational Tools. J. Bioinform. Seq. Anal..

[52] Minetoki T., Tsuboi H., Koda A. (2013). Development of High Expression System with the Improved Promoter Using the Cis-Acting Element in Aspergillus Species. J. Biol. Macromol..

[53] Dave K., Punekar N.S. (2011). Utility of *Aspergillus Niger* Citrate Synthase Promoter for Heterologous Expression. J. Biotechnol..

[54] Ortiz-Urquiza A., Keyhani N.O. (2015). Stress Response Signaling and Virulence: Insights from Entomopathogenic Fungi. Curr. Genet..

[55] Gao J., Sun Y., Jin W., Zhang F., Zhou M., Song X. (2024). Methyl Jasmonate Mitigates Fusarium Graminearum Infection in Wheat by Inhibiting Deoxynivalenol Synthesis. Physiol. Plant..

[56] Yamamoto A., Mizukami Y., Sakurai H. (2005). Identification of a Novel Class of Target Genes and a Novel Type of Binding Sequence of Heat Shock Transcription Factor in Saccharomyces Cerevisiae. J. Biol. Chem..

[57] Casas-Flores S., Rios-Momberg M., Bibbins M., Ponce-Noyola P., Herrera-Estrella A. (2004). BLR-1 and BLR-2, Key Regulatory Elements of Photoconidiation and Mycelial Growth in Trichoderma Atroviride. Microbiology.

[58] Kim S.R., Choi J.L., Costa M.A., An G. (1992). Identification of G-Box Sequence as an Essential Element for Methyl Jasmonate Response of Potato Proteinase Inhibitor II Promoter. Plant Physiol..

[59] Menkens A.E., Schindler U., Cashmore A.R. (1995). The G-Box: A Ubiquitous Regulatory DNA Element in Plants Bound by the GBF Family of bZIP Proteins. Trends Biochem. Sci..

[60] Liu Q.J., Yi J., Wu G., Gao X., Jia B., Tang P., He M., Shi K., Zeng S., Li J. (2022). Identification, culture, and pathogenicity of an entomopathogenic fungus from the larvae of Xylotrechus quadripes. Sci. West. For..

[61] Fu-chu H. (2006). Integrated Nr Database in Protein Annotation System and Its Localization. Comput. Eng..

[62] Boeckmann B., Bairoch A., Apweiler R., Blatter M.-C., Estreicher A., Gasteiger E., Martin M.J., Michoud K., O’Donovan C., Phan I. (2003). The SWISS-PROT Protein Knowledgebase and Its Supplement TrEMBL in 2003. Nucleic Acids Res..

[63] Kanehisa M., Goto S., Kawashima S., Okuno Y., Hattori M. (2004). The KEGG Resource for Deciphering the Genome. Nucleic Acids Res..

[64] Tatusov R.L., Galperin M.Y., Natale D.A., Koonin E.V. (2000). The COG Database: A Tool for Genome-Scale Analysis of Protein Functions and Evolution. Nucleic Acids Res..

[65] Conesa A., Götz S., García-Gómez J.M., Terol J., Talón M., Robles M. (2005). Blast2GO: A Universal Tool for Annotation, Visualization and Analysis in Functional Genomics Research. Bioinformatics.

[66] Ashburner M., Ball C.A., Blake J.A., Botstein D., Butler H., Cherry J.M., Davis A.P., Dolinski K., Dwight S.S., Eppig J.T. (2000). Gene Ontology: Tool for the Unification of Biology. The Gene Ontology Consortium. Nat. Genet..

[67] Eddy S.R. (1998). Profile Hidden Markov Models. Bioinformatics.

[68] Finn R.D., Coggill P., Eberhardt R.Y., Eddy S.R., Mistry J., Mitchell A.L., Potter S.C., Punta M., Qureshi M., Sangrador-Vegas A. (2016). The Pfam Protein Families Database: Towards a More Sustainable Future. Nucleic Acids Res..

[69] Emms D.M., Kelly S. (2019). OrthoFinder: Phylogenetic Orthology Inference for Comparative Genomics. Genome Biol..

[70] Edgar R.C. (2022). Muscle5: High-Accuracy Alignment Ensembles Enable Unbiased Assessments of Sequence Homology and Phylogeny. Nat. Commun..

[71] Castresana J. (2000). Selection of Conserved Blocks from Multiple Alignments for Their Use in Phylogenetic Analysis. Mol. Biol. Evol..

[72] Tamura K., Stecher G., Kumar S. (2021). MEGA11: Molecular Evolutionary Genetics Analysis Version 11. Mol. Biol. Evol..

[73] Blum M., Andreeva A., Florentino L.C., Chuguransky S.R., Grego T., Hobbs E., Pinto B.L., Orr A., Paysan-Lafosse T., Ponamareva I. (2025). InterPro: The Protein Sequence Classification Resource in 2025. Nucleic Acids Res..

[74] Lombard V., Golaconda Ramulu H., Drula E., Coutinho P.M., Henrissat B. (2014). The Carbohydrate-Active Enzymes Database (CAZy) in 2013. Nucleic Acids Res..

[75] Letunic I., Khedkar S., Bork P. (2021). SMART: Recent Updates, New Developments and Status in 2020. Nucleic Acids Res..

[76] Lu S., Wang J., Chitsaz F., Derbyshire M.K., Geer R.C., Gonzales N.R., Gwadz M., Hurwitz D.I., Marchler G.H., Song J.S. (2020). CDD/SPARCLE: The Conserved Domain Database in 2020. Nucleic Acids Res..

[77] Walker J.M. (2005). The Proteomics Protocols Handbook.

[78] Horton P., Park K.-J., Obayashi T., Fujita N., Harada H., Adams-Collier C.J., Nakai K. (2007). WoLF PSORT: Protein Localization Predictor. Nucleic Acids Res..

[79] Bailey T.L., Johnson J., Grant C.E., Noble W.S. (2015). The MEME Suite. Nucleic Acids Res..

[80] Paysan-Lafosse T., Andreeva A., Blum M., Chuguransky S.R., Grego T., Pinto B.L., Salazar G.A., Bileschi M.L., Llinares-López F., Meng-Papaxanthos L. (2024). The Pfam Protein Families Database: Embracing AI/ML. Nucleic Acids Res..

[81] Waterhouse A., Bertoni M., Bienert S., Studer G., Tauriello G., Gumienny R., Heer F.T., de Beer T.A.P., Rempfer C., Bordoli L. (2018). SWISS-MODEL: Homology Modelling of Protein Structures and Complexes. Nucleic Acids Res..

